# Application Value of PET/CT and MRI in the Diagnosis and Treatment of Patients With Synchronous Multiple Pulmonary Ground-Glass Nodules

**DOI:** 10.3389/fonc.2022.797823

**Published:** 2022-02-23

**Authors:** Shaonan Xie, Shaoteng Li, Huiyan Deng, Yaqing Han, Guangjie Liu, Qingyi Liu

**Affiliations:** ^1^ Department of Thoracic Surgery, The Fourth Hospital of Hebei Medical University, Shijiazhuang, China; ^2^ Department of Diagnostic Radiology, The People’s Hospital of Xingtai, Xingtai, China; ^3^ Department of Pathology, The Fourth Hospital of Hebei Medical University, Shijiazhuang, China

**Keywords:** synchronous multiple ground-glass nodules (SMGGNs), positron-emission tomography and computed tomography (PET/CT), magnetic resonance imaging (MRI), lung cancer, adenocarcinoma

## Abstract

**Background:**

Synchronous multiple ground-glass nodules (SMGGNs) in synchronous multiple lung cancers are associated with specific imaging findings. It is difficult to distinguish whether multiple nodules are primary tumors or metastatic lesions in the lungs. The need for PET/CT and contrast-enhanced brain MRI for these patients remains unclear. This study investigated the necessity of these two imaging examinations for SMGGN patients by means of retrospective analysis.

**Methods:**

SMGGN patients who were diagnosed and treated in our hospital from October 2017 to May 2020 and underwent whole-body PET/CT(Cranial excepted) and/or contrast-enhanced brain MRI+DWI were enrolled in this study. We analyzed the imaging and clinical characteristics of these patients to evaluate SMGGN patients’ need to undergo whole-body PET/CT and brain MRI examination.

**Results:**

A total of 87 SMGGN patients were enrolled. 51 patients underwent whole-body PET/CT examinations and did not show signs of primary tumors in other organs, metastatic foci in other organs, or metastasis to surrounding lymph nodes. 87 patients underwent whole-brain MRI, which did not reveal brain metastases but did detect an old cerebral infarction in 23 patients and a new cerebral infarction in one patient. 87 patients underwent surgical treatment in which 219 nodules were removed. All nodules were diagnosed as adenocarcinoma or atypical adenomatous hyperplasia. No lymph node metastasis was noted.

**Conclusion:**

For SMGGN patients, PET/CT and enhanced cranial MRI are unnecessary for SMGGNs patients, but from the perspective of perioperative patient safety, preoperative MRI+DWI examination is recommended for SMGGNs patients.

## Introduction

The incidence of synchronous multiple lung cancers (SMLCs) accounts for approximately 0.2% of all lung cancers, but the incidence of SMLCs has tended to gradually increase worldwide (1). The reason for this situation may be due to the popularization of low-dose computed tomography (LDCT) and high-resolution CT (HRCT), as well as its promotion and application in early lung cancer screening. Particularly, LDCT and HRCT can be used to find ground-glass nodules (GGNs) in the lungs that cannot be found on traditional chest X-ray (2). However, lung cancer patients with multiple lung lesions have long been difficult to classify due to the inability to distinguish between independent primary tumors and lung cancer with intrapulmonary metastasis, in addition, several patterns of radiological expression are associated with SMLCs (3). To provide better clarity, the 8^th^ edition of the tumor–node–metastasis (TNM) classification for lung cancer developed by the International Association for the Study of Lung Cancer (IASLC) shows that lung cancers that manifest as multiple foci in imaging studies are classified into four categories: secondary primary lung cancer, isolated tumor nodules (intrapulmonary metastasis), multiple GGNs, and pneumonic-type lung adenocarcinoma (4, 5).

The National Comprehensive Cancer Network (NCCN) Clinical Practice Guidelines in Oncology (NCCN guidelines, the 2nd edition, 2020) recommend performing positron-emission tomography/computed tomography (PET/CT) and contrast-enhanced brain magnetic resonance imaging(MRI) for patients with multiple-nodule lung cancer (6). However, the guidelines do not specify anything about the type of multifocal lung cancer. The NCCN guidelines suggest using PET/CT as a preoperative evaluation for patients with multifocal lung cancer to assess whether there is mediastinal lymph node metastasis or distant metastasis (6). Due to the deficiencies of PET/CT in brain imaging, contrast-enhanced brain MRI is used to assess whether patients have neurological metastases (5).

Although the incidence of synchronous multiple GGNs (SMGGNs) has not been quantified, it is becoming more common as one of the imaging manifestations of SMLCs. It is mostly considered to be multiple early primary lung adenocarcinomas or precancerous lesions. Whether patients with SMGGNs can benefit from preoperative PET/CT and brain MRI needs further study. Therefore, to understand the effectiveness and necessity of these modalities in these patients, we conducted a retrospective analysis and evaluation of patients with SMGGNs who underwent routine PET/CT(Cranial excepted) and contrast-enhanced brain MRI+dispersion weighted images sequences(DWI) before surgery to determine whether there was mediastinal lymph node metastasis or organ metastasis, including determining whether SMGGNs were intrapulmonary metastasis.

## Methods

This retrospective study was reviewed and approved by the Ethics Committee of the Fourth Hospital of Hebei Medical University. Since the data of this study are retrospective and anonymous, no informed consent is required.

### Enrollment of Patients

SMGGN patients who were diagnosed and treated in the Department of Thoracic Surgery in our hospital from October 2017 to May 2020 and underwent whole-body PET/CT(Cranial excepted) and contrast-enhanced brain MRI+DWI examinations were enrolled in this study. SMGGNs include multiple pure GGNs (pGGNs) and multiple mixed GGNs (mGGNs). pGGNs are seen as focal ground-glass shadows on the lung window on CT, and the nodules must not contain solid components that can block the structure of blood vessels or bronchi (7). mGGNs are GGNs that show up as shadows and contain solid components that block the structure of blood vessels or bronchi. In this study, the patients were selected based on their thin-slice CT images and the above definitions. The CT diagnosis of SMGGNs was defined as two or more GGNs shown on the images in which the maximum diameter of the solid component of mGGNs is not greater than 5 mm. The patients were diagnosed with precancerous lesions or early lung cancer if they had a relevant medical history. Two radiologists and two thoracic surgeons evaluated and compared the CT images longitudinally and reached an agreement on patient enrollment.

### Image Acquisition and Analysis

All scanning was performed on a Gemini GXL 16‐slice PET/CT system (Philips) with ^18^F-fluorodeoxyglucose (FDG) (radiochemical identity/purity > 95%) provided by Andico. The patient had fasted for more than 6 hours. The patient’s height and weight and level of fasting blood glucose (<6.1 mmol/L) were measured. In the resting state, 222-492.1 MBq (6-13.3 mCi) ^18^F-FDG was injected *via* the dorsal vein of the hand. PET/CT was performed 50-60 minutes after injection, during which the patient was resting in a dark room. The patient was in the supine position with both hands on the head. Multislice spiral CT scan was performed first. The scan range was from the neck to the upper segment of the femur. The scan conditions were as follows: voltage 120 kV, current 160 mA, slice thickness 5 mm, interslice gap 5 mm, matrix 512 × 512, helical pitch 0.813, and single rotation time of the tube 0.5 s. The patient was asked to breathe calmly to ensure the scanning images to synchronize with the PET images. Then, the PET scan was performed in the 3D acquisition mode with an acquisition speed of 2.5 min/frame for a total of 8-10 frames. PET images of PET/CT were three-dimensionally reconstructed using the 3D line of response reconstruction algorithm. At the same time, CT data were used for attenuation compensation of the PET images. Both the slice thickness and interslice gap were 5 mm. During a breath-hold, a thin-slice CT scan was carried out on all GGNs in the lungs for reconstruction, with a slice thickness of 0.8 mm. When the pulmonary nodules were suspected of being malignant on PET or CT images, delayed PET scan of the chest was performed 120 minutes after injection of ^18^F-FDG.

Contrast-enhanced brain MRI was performed by a 1.5- or 3.0-T MRI scanner (GE), with the parameters as follows: axial FSE T2WI/FLAIR (repetition time (TR), 9000 ms; echo time (TE), 96 ms; number of excitations (NEX), 1; slice thickness, 5 mm), intravenous contrast agent Gd-DTPA (TR, 1700 ms; TE, 2.32 ms; NEX, 1; slice thickness, 5 mm) for axial and sagittal FSE T1WI scanning; DWI sequence (TR, 4100 ms; TE, 64 ms; NEX, 1; slice thickness, 5 mm); and MRA (TR, 22 ms; TE, 3.67 ms; NEX, 1; slice thickness, 5 mm).

### Treatment and Pathological Staging

Pathological diagnosis and staging were done in patients undergoing surgical treatment. The postoperative pathological diagnosis was performed according to the standards developed by IASLC/American Thoracic Society/European Respiratory Society classification. Pathological staging was based on the 8th edition of the IASLC lung cancer staging system. Molecular pathological analysis on surgical specimens was performed to investigate the mutation status of the epidermal growth factor receptor (*EGFR*) gene.

## Results

### Patient Selection

A total of 109 patients who were diagnosed and treated in the Department of Thoracic Surgery in our hospital from October 2017 to May 2020. 87 patients underwent surgical treatment. All of them underwent enhanced MRI+DWI of the head, and 51 of them underwent whole-body PET/CT(Cranial excepted). All 51 whole-body PET/CT examinations showed no signs of primary tumors in other organs, metastatic foci of other organs, or metastasis to surrounding lymph nodes (no abnormal high FDG uptake was found). All patients underwent contrast-enhanced brain MRI+DWI scan, and it did not reveal brain metastases, though it did detect an old cerebral infarction in 23 patients and new cerebral infarction in one patient by DWI sequences.

**Figure 1:**
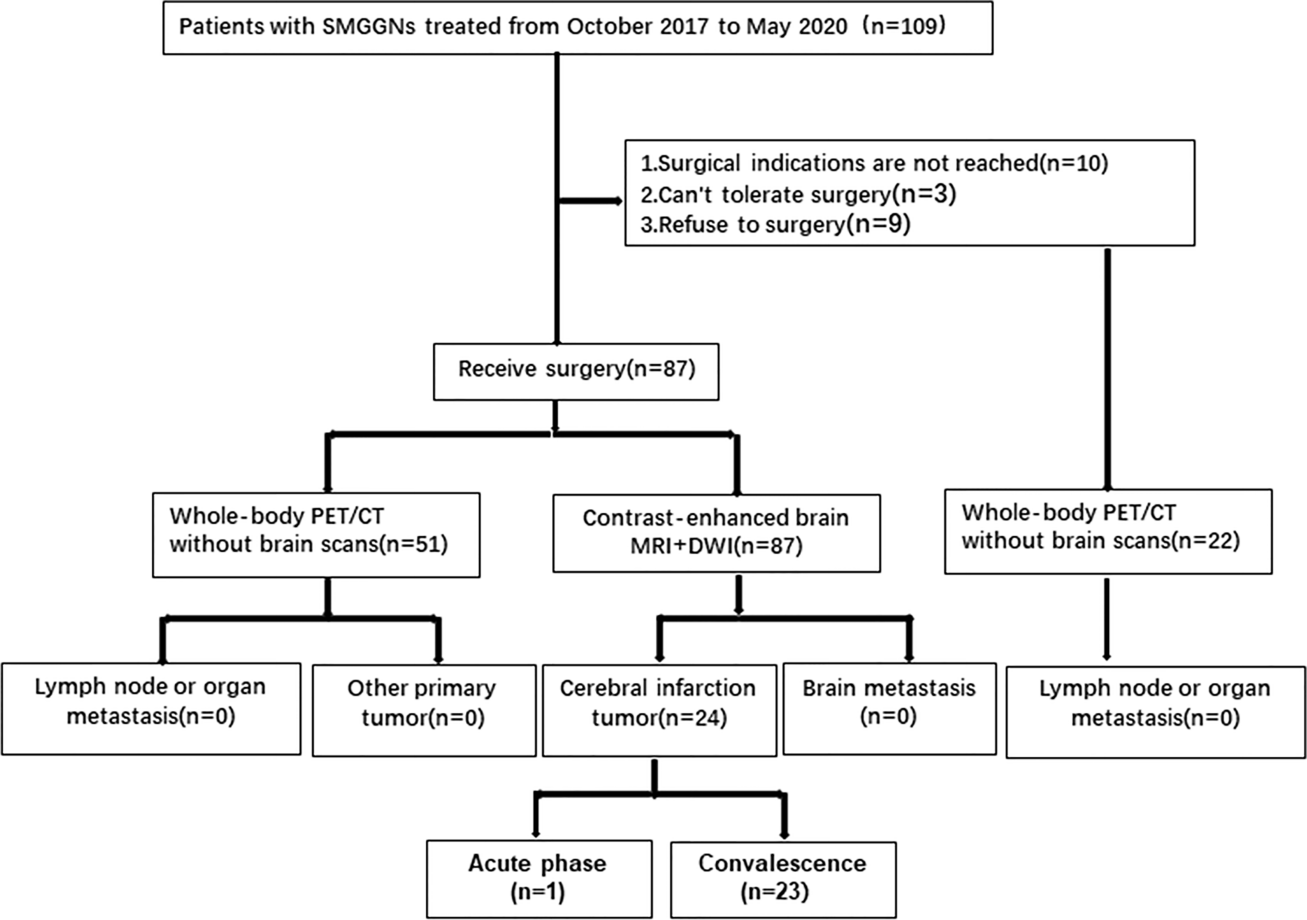
Flow chat of SMGGN patient enrollment and examination.

22 patients did not undergo surgical treatment. 10 patients’ dominant nodules did not meet surgical indications, 3 patients could not tolerate surgery because of underlying disease, and 9 patients refused surgical treatment. All of them underwent whole-body PET/CT(Cranial excepted) too. And All of the whole-body PET/CT examinations showed no signs of primary tumors in other organs, metastatic foci of other organs, or metastasis to surrounding lymph nodes (no abnormal high FDG uptake was found).No patients with SMGGNs abandoned surgery because they were considered for intrapulmonary metastasis (**Figure 1**).

### Characteristics and Imaging Data of the Patient

The median age of the 87 SMGGN patients at onset was 58 years (43-75 years). There were 26 men (29.9%) and 61 women (70.1%). A total of 351 GGNs were observed in the whole group of patients (nodules less than 5 mm were not included). The number of GGNs in each patient was two in 51 patients, 3-5 in 19 patients, 6-10 in 6 patients, and more than 10 in 11 patients. The diameter of the largest nodule on CT was ≤ 10.0 mm in 13 patients, > 10.0 mm and ≤ 20 mm in 42 patients, > 20.0 mm and ≤ 30 mm in 29 patients, and > 30 mm in 3 patients (**Table 1**).

**Table 1:** Clinical characteristics of 87 patients.

Variable/characteristic	Result/No. of patients (%)
**Median age, years**	58 (range, 43-75)
**Sex**	
**Male**	26 (29.9)
**Female**	61 (70.1)
**Smoking history**	
**Yes**	18 (20.7)
**No**	69 (79.3)
**Initial symptoms**	
**Asymptomatic**	69 (79.3)
**Cough**	14 (16.1)
**Other symptoms**	4 (4.6)
**No. of GGNs per patient**	477
**2**	51 (58.6)
**3-5**	19 (21.8)
**6-10**	6 (6.9)
**>10**	11 (12.6)
**Tumor size of dominant lesion on CT**	
**≤10 mm**	13 (14.9)
**>10 mm, ≤20 mm**	42 (48.3)
**>20 mm, ≤30 mm**	29 (33.3)
**>30 mm**	3 (3.4)
**Average SUV_max_ of dominant lesion on PET/CT**	1.1

### Surgical and Pathological Outcomes After Surgery

63 patients underwent surgery directly after completing preoperative examination. Of the 23 patients diagnosed with chronic cerebral infarction,14 patients underwent surgery after short-term (7 days) antiplatelet aggregation therapy. 9 patients underwent surgery after cranial MRA and carotid ultrasound to screen blood vessels and control for risk factors. The patient with a new cerebral infarction was treated for cerebral infarction and underwent surgery 6 months later. No cerebrovascular accident occurred in all patients during perioperative period.

A total of 219 GGNs were resected in 87 patients. Postoperative pathology showed invasive adenocarcinoma (AC) in 75 GGNs, minimally invasive adenocarcinoma (MIA) in 47 GGNs, adenocarcinomas *in situ* (AIS) in 47 GGNs, and atypical adenomatous hyperplasia (AAH) in 50 GGNs. There were no cases of pleural invasion or vascular tumor thrombus, and no metastasis was found in the sampled lymph nodes. One GGN was removed in 19 patients, multiple GGNs were removed from the same lobe in five patients, GGNs in different lobes on the same side were removed in 36 patients, and bilateral GGNs were removed in 27 patients at the same time or in stages. The number of the resected GGNs was one in 19 patients, two in 36 patients, three in 17 patients, four in seven patients, five in four patients, six in one patient, seven in two patients, and nine in one patient (**Table 2**).

**Table 2:** Pathological and molecular-biological characteristics of 87 patients.

Variable/characteristic	Result/No. of patients (%)
**Surgical procedure**	87 (100)
**pGGNs resected**	219
**Operation**	
**Ipsilateral**	60 (69.0)
**Bilateral**	27 (31.0)
**Histological type (219 pGGNs)**	
**AAH**	50 (22.8)
**AIS**	47 (21.5)
**MIA**	47 (21.5)
**AC**	75 (34.2)
**Pathological lymph nodal metastasis**	
**No (N_0_)**	87 (100)
**Yes (N_1-2_)**	0 (0)
**Molecular pathology (194 pGGNs)**	
***EGFR* wild-type**	78 (42.7)
***EGFR* mutated**	109 (58.3)
**21L858R**	54 (27.8)
**21L861Q**	3 (1.5)
**19deletion**	44 (22.7)
**18G719X**	2 (1.0)
**20T790M**	2 (1.0)
**20S768I**	1 (0.5)
**21L858R+2L62R**	1 (0.5)
**21L858R+20T790M**	1 (0.5)
**19deletion+21L858R**	1 (0.5)

**Figure 2:**
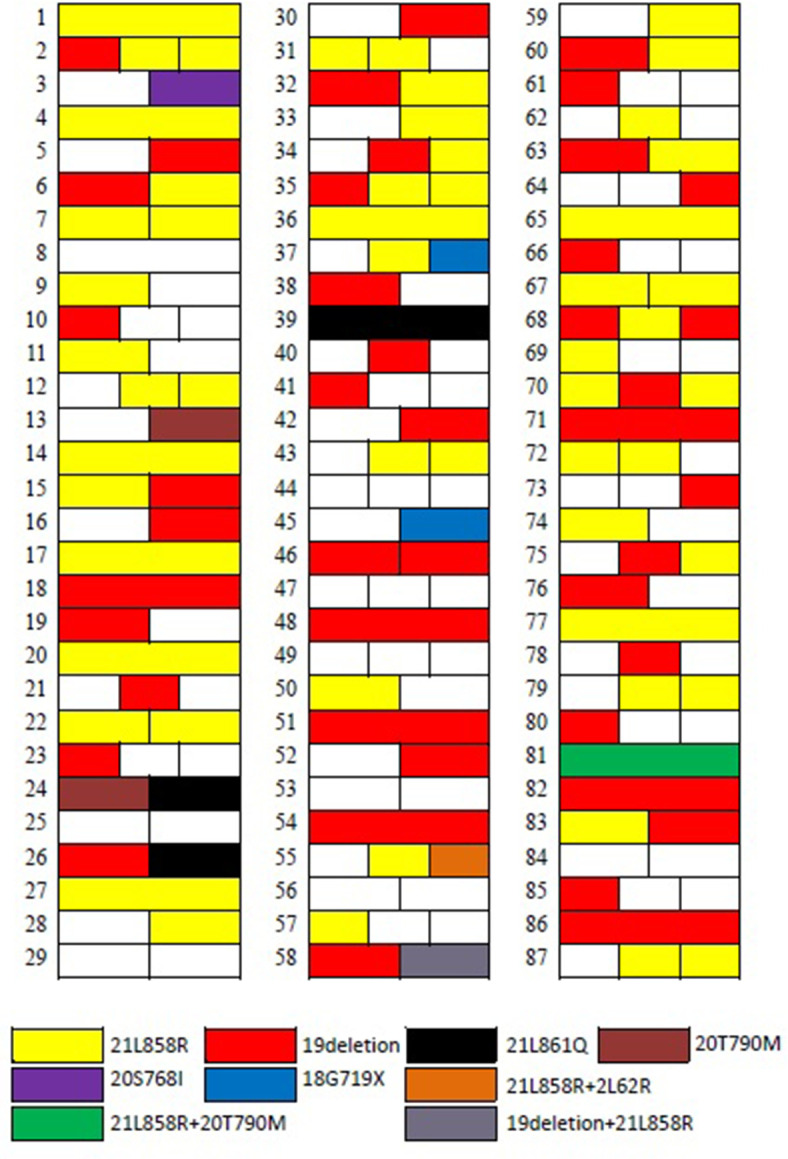
The distribution of EGFR mutations in 87 patients.

In patients with more than three (including five) GGNs removed, the largest three GGNs were tested for *EGFR* gene mutation. Among the 68 patients with multiple GGNs removed, 57 patients had one of various *EGFR* mutations (83.8%, 57/68) in their lung GGNs (**Figure 2**).

## Discussion

We conducted this retrospective analysis to evaluate the necessity of preoperative whole-body PET/CT and contrast-enhanced brain MRI in patients with SMGGNs. Although false-positive uptake of ^18^F-FDG is often seen in daily PET/CT studies (8), none of our 51 patients undergoing PET/CT had other primary or secondary lesions in distant organs or metastasis to mediastinal lymph nodes. In all patients undergoing surgery, no metastatic lymph nodes were found in postoperative pathological examination. The preoperative screening section of the NCCN Guidelines for non-small cell lung cancer in the United States recommends that patients with SMLC undergo a whole-body PET/CT and a contrast-enhanced MRI examination of the head before surgery (9). For patients with multiple pGGNs, whether these two examinations are required before surgery is inconclusive, and no scientific consensus has been reached.

On CT images, lung nodules can be divided into solid nodules (SNs), part-solid nodules (PSNs) or mGGNs, and nonsolid nodules or pGGNs. Both pGGNs/NSNs and mGGNs/PSNs are called subsolid nodules (SSNs) (10–12). We added an additional high-resolution CT scan at breath-hold for patients who had GGNs identified on the thin-slice CT in the subsequent PET/CT scan, so the diagnosis of pGGN is reliable.

Lung GGNs that persist in CT scans is considered an imaging manifestation of early lung adenocarcinoma or precancerous lesions (13). According to the new lung adenocarcinoma staging system (4), GGNs can show a growth pattern of attachment to the alveolar wall under the microscope. This indicates a lower invasiveness. This growth pattern has little effect on alveolar ventilation. This feature of GGNs can be observed on CT images (14). The 219 nodules we resected were pathologically diagnosed as invasive adenocarcinoma, minimally invasive adenocarcinoma, adenocarcinoma *in situ*, or lung atypical adenomatous hyperplasia after surgery. Lung adenocarcinoma is the most common pathological type of lung cancer and is associated with mutations in a variety of oncogenes. The most commonly mutated genes include *EGFR*, *ALK*, *BRAF*, and *KRAS*. We performed *EGFR* mutation detection on 187 of these nodules, and 109 *EGFR* mutations were detected, for a mutation rate of 58.3%.

The cause of GGNs still needs to be investigated. GGNs are somewhat different from typical lung cancer. There is no obvious relationship between the occurrence of GGNs and smoking (a carcinogen). Most GGN patients do not smoke. The occurrence and development of GGNs are relatively slow, mostly in the peripheral part of the lung, and multiple primary lesions may be present. Household air pollution may be related to the incidence of GGNs. Household air pollution includes exhaust gas from burning solid fuels for heating and cooking and oil fumes that come from cooking (15). In our study, females (61/87) and nonsmoking patients (69/87) patients accounted for most of the enrolled patients.

Like other SMLCs, SMGGNs cause confusion for clinicians. It is hard to tell whether an SMGGNs are a lung metastasis of the same primary cancer or are multiple primary lung cancers (MPLCs) of different origins. Moreover, this confusion has not been resolved by advancements in pathology, because the SMGGNs usually have the same histological type, even if they have different growth patterns (16). According to the traditional definition, MPLCs with the same histological results must be evaluated according to the following criteria: 1. The histological origin is carcinoma *in situ*. 2. No lymph nodes in the conventional lymph node metastasis pathways are involved. 3. There is no extrathoracic metastasis (17, 18). In our study, we found no signs of metastasis on any PET/CT images, including lymph node metastasis and organ metastasis. These findings confirm that the multiple pGGNs were MPLCs with multiple primary lesions. In the Fleischner Society and IASLC statements, SMGGNs are considered to be the early stages of MPLC.

In addition to identifying and classifying SMGGNs based on histopathological findings, researchers and clinicians have also used molecular biology methods. The molecular biology test results of tissue specimens strongly support the view that they are all independent primary tumors (19, 20). In our study, among the 68 patients with multiple nodules removed, 57 patients had lung nodules with *EGFR* mutation status (83.8%, 57/68), confirming the above results. Base on this, we believe that we believe that for a molecular biology point of view, all SMGGNs are independent primary tumors, it is invalid to use PET/CT to determine whether the SMGGNs are metastatic nodules or to look for other primary tumor. However, Li et al. (21) published an article about the occurrence of intrapulmonary metastases in the form of multiple GGNs. They performed whole-exome sequencing on each of the removed nodules in two patients with multiple GGNs in the lungs. They found in each of these two patients that two GGNs shared multiple rare nonsynonymous and synonymous mutations, which strongly suggested that they were intrapulmonary metastases. In contrast, the remaining GGNs showed different clonal origins. The reason for the early metastasis of GGNs may be the dissemination of tumor cells in the alveolar cavity. Although this new metastasis model of lung cancer has been well accepted, whether there are metastases in multiple GGNs in the lungs, especially multiple GGNs in the bilateral lungs, still needs to be studied and verified.

Lesions (including single lesions and multiple lesions) that appear as pure GGNs on CT images and tumors with growth patterns of attachment to the wall show indolent biological behavior and are associated with a relatively good prognosis (22, 23). Based on the multiple origins of multiple GGNs in the lungs confirmed by our research and the absence of lymph node metastasis, we have reason to believe that for patients with multiple GGNs in the lungs, limited lung resection with close follow-up should be recommended over lobectomy to observe the remaining nodules after surgery. This is the best diagnostic and treatment strategy for patients with multiple GGNs in the lung. Next-generation gene sequencing technology can be used for whole-genome sequencing, whole-exome sequencing, or target gene sequencing on surgically resected specimens to analyze whether multiple GGNs have the same origin (24). This is necessary for the overall management of the disease in patients with multiple GGNs.

It has been report (25, 26) that the sensitivity of crania enhanced MRI in screening for brain metastasis of lung cancer is not inferior to or even higher than that of whole-body PET/CT, so in our study cranial enhanced MRI was used to screen for intracranial metastasis. Although the preoperative MRI of all 87 patients did not show intracranial metastases, but combined with DWI sequence, it did reveal an old cerebral infarction in 23 patients and a new cerebral infarction in one patient. The new onset of cerebral infarction indicates the patient is in a period of hemodynamic instability, so cerebrovascular accident is more likely to recur during the perioperative period after video-assisted thoracoscopic lung resection (27). Patients with old cerebral infarction and abnormal cerebrovascular stenosis are more likely to have cerebrovascular accidents when undergoing lung (especially upper lobe) resection than the healthy population (28). Compared with PET/CT, head MRI+DWI has significant advantages in the diagnosis of cerebral infarction (29). Therefore, we believe that for patients with multiple GGNs, preoperative contrast-enhanced brain MRI is necessary.

This study has some limitations. First, the sample size of this study was relatively small. The high cost of PET/CT examinations made it hard to enroll many cases. Second, the study is limited by its retrospective design, which may have caused selection bias. Prospective studies should evaluate the practicality of preoperative PET/CT and brain MRI. Finally, we only included patients with multiple pGGNs and mGGNs with solid components ≤ 5 mm and excluded patients with mGGNs with solid components > 5 mm. Since mGGNs with a solid component > 5 mm are more invasive, PET/CT and brain MRI in SMGGNs of patients with a solid component > 5 mm before surgery require further study.

## Conclusion

Whole-body PET/CT and contrast-enhanced brain MRI didn’t provide additional information to determine whether patients with SMGGNs had intrapulmonary metastases and the presence of lymph node and organ metastases., but the DWI sequence can help us find some surgical risk factors before surgery, screen some patients who are not suitable for surgery, and intervene in advance for those patients who still have the chance of surgery, so as to reduce the perioperative surgical risk of patients. Based on the results of our study, we believe that PET/CT and enhanced cranial MRI are unnecessary for SMGGNs patients, but from the perspective of perioperative patient safety, preoperative MRI+DWI examination is recommended for SMGGNs patients. Of course, this conclusion is based on our retrospective study, and we expect RCT studies to prove this conclusion.

## Data Availability Statement

The original contributions presented in the study are included in the article/**Supplementary Material**. Further inquiries can be directed to the corresponding author.

## Ethics Statement

Ethical review and approval was not required for the study on human participants in accordance with the local legislation and institutional requirements. Written informed consent for participation was not required for this study in accordance with the national legislation and the institutional requirements. Written informed consent was not obtained from the individual(s) for the publication of any potentially identifiable images or data included in this article.

## Author Contributions

SX: Conceptualization, Data curation, Writing - original draft. SL: Data curation. HD: Formal analysis, Writing - review & Editing. YH: Data curation; Formal analysis. GL: Investigation; Methodology. QL: Conceptualization, Funding acquisition, Writing - review & Editing. All authors contributed to the article and approved the submitted version.

## Funding

This work was supported by Medical science research project of Hebei province (Grant No. 20201076), The programme of the government funding Clinical Excellence of Hebei province (2019 Grant No. 139).

## Conflict of Interest

The authors declare that the research was conducted in the absence of any commercial or financial relationships that could be construed as a potential conflict of interest.

## Publisher’s Note

All claims expressed in this article are solely those of the authors and do not necessarily represent those of their affiliated organizations, or those of the publisher, the editors and the reviewers. Any product that may be evaluated in this article, or claim that may be made by its manufacturer, is not guaranteed or endorsed by the publisher.
