# Supplementary figures and images for: Application Value of PET/CT and MRI in the Diagnosis and Treatment of Patients With Synchronous Multiple Pulmonary Ground-Glass Nodules
Source: Front Oncol. 2022 Feb 23;12:797823. doi: 10.3389/fonc.2022.797823 (PMC8905144; doi:10.3389/fonc.2022.797823)

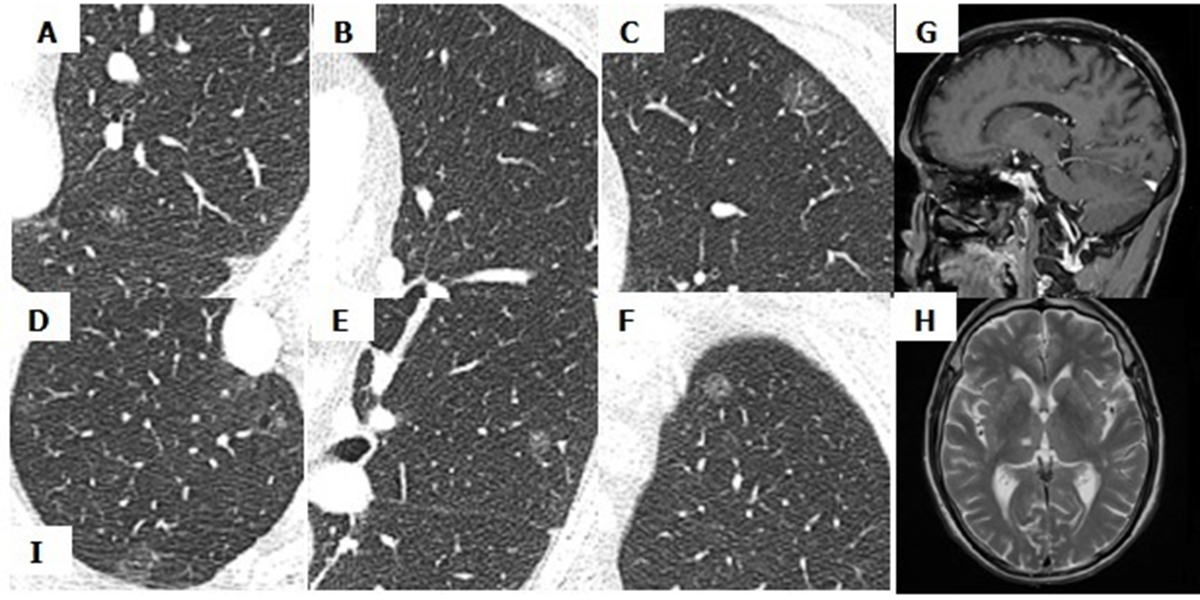

Supplement: Supplementary file 1 [file Image_1.tif]

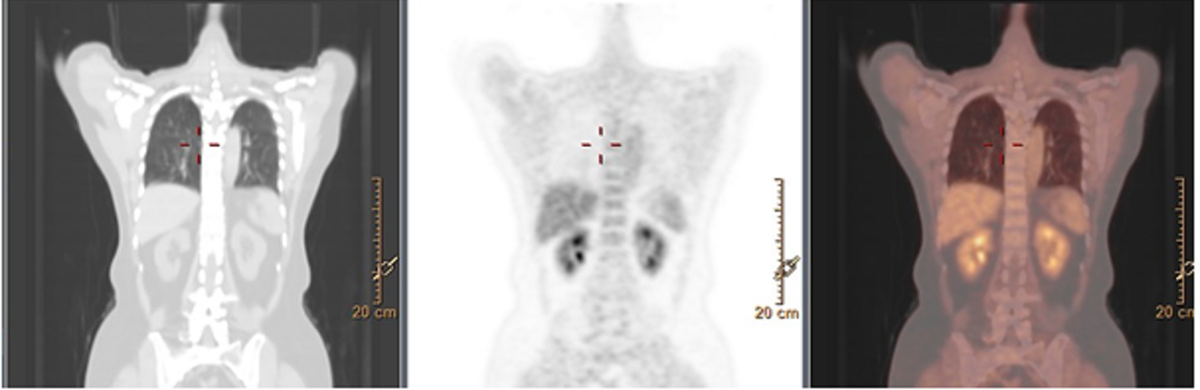

Supplement: Supplementary file 2 [file Image_2.tif]

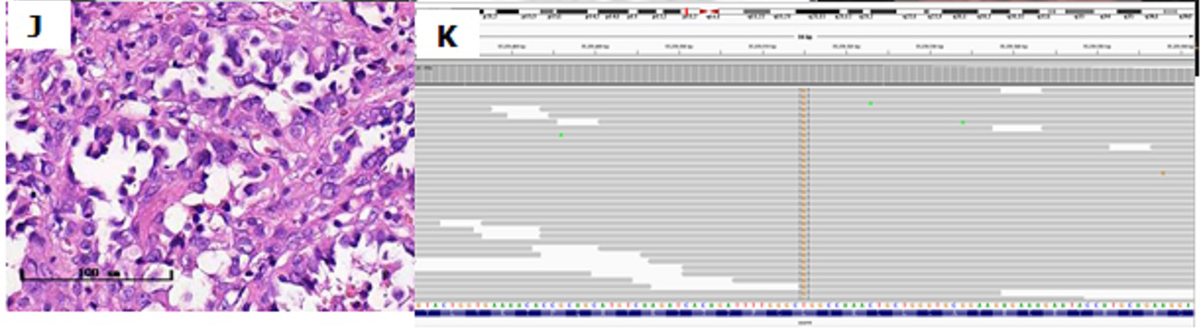

Supplement: Supplementary file 3 [file Image_3.tif]
